# Loading and release of cancer chemotherapy drugs utilizing simultaneous temperature and pH-responsive nanohybrid

**DOI:** 10.1186/s40360-021-00508-8

**Published:** 2021-07-14

**Authors:** Mohammad Dahri, Hossein Akbarialiabad, Ahmad Miri Jahromi, Reza Maleki

**Affiliations:** 1 Computational Biology and Chemistry Group (CBCG), Universal Scientific Education and Research Network (USERN), Tehran, Iran; 2 Student Research Committee, School of Medicine, Shiraz University of Medical Sciences, Shiraz, Iran

**Keywords:** Cancer, Doxorubicin, Paclitaxel, Dimethyl acrylamide-trimethyl chitosan, Molecular dynamic, pH-responsive

## Abstract

**Background:**

Recently, the development of nanocarriers and the improvement of their biochemical properties have became of great importance. Single-walled carbon nanotubes (SWCNT) have many applications in drug delivery systems (DDS) as a common carbon-based structure. In the current work, the penetration, co-loading, and co-release of Doxorubicin (DOX) and Paclitaxel (PAX), as two cancer chemotherapy agents, were investigated using a novel modified copolymer with functionalized SWCNT.

**Results:**

This study proposes a dual-responsive smart carrier that is sensitive to pH and temperature. The carrier consists of functionalized SWNT and Dimethyl acrylamide-trimethyl chitosan (DMAA-TMC) grafting on SWCNT. This suggested carrier was investigated by utilizing molecular simulations. Interaction energies between DOX, PAX, and carrier as well as the affinity of drugs to the nanocarrier were studied. The energy analysis of drug release and adsorption presented that DOX and PAX delivery using this carrier is selective and sensitive at healthy and cancerous conditions. The attraction of DMAA-TMC, as a biodegradable and biocompatible copolymer, with SWCNT showed that degradation mechanism in acidic environment deformed the copolymer. This leads to a smart release mechanism in an acidic cancerous tissue. Additionally, it improves hydrophilicity, optimum nano-particle size, and cell cytotoxicity concerns.

**Conclusions:**

The simulation results manifested a significant contribution of DMAA-TMC in the adsorption and release of cancer chemotherapy drugs in normal and neoplastic tissues. The interaction of copolymer also improves the biocompatibility and biodegradability of the SWCNT. Smart drug delivery carrier can be a valuable nanohybrid for loading, transporting, and releasing of cancer chemotherapy drugs.

**Graphical abstract:**

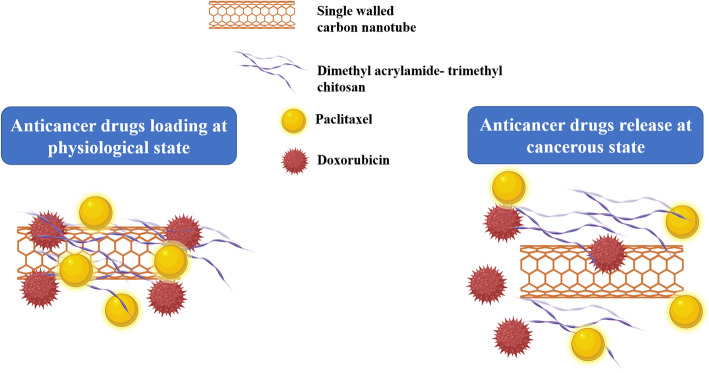

## Background

Cancer is a category of disease implicating abnormal cell growth that invades or spreads to other destinations of the body [1, 2]. Based on the tissue origin, solid malignancies is mainly divided into the following two types [3, 4]:

1. Carcinoma of epithelial cell origin (such as adenocarcinoma) [5]
2. Sarcoma of non-epithelial cell origin (such as liposarcoma and osteosarcoma)

The characteristics of tumor cells are varied, and some of these characteristics may be fatal. Some of these include uncontrolled proliferation or delay in apoptosis, decreased cell differentiation, the ability to attack adjacent tissue, and the ability to establish new cell proliferation at inappropriate sites known as metastasis [4]. However, not all types of cancers metastasize.

There are many methods to treat cancer. Chemotherapy, as a usual choice, has many obstacles. The failure of chemotherapy to have successful selective treatment is a significant problem in clinical oncology [6, 7]. The resistance of cancerous cells by complex immune escaping mechanisms and specific cancerous microenvironment conditions encourages pursuing new strategies [8–11]. There are many chemotherapic drugs; among them, Paclitaxel (PAX) and Doxorubicin (DOX) are conventional cancer chemotherapy drugs. DOX, as an antibiotic, and PAX as a herbal medicine, are two significant natural sources of anticancer drugs in chemotherapy [12, 13]. DOX combats with the production of proteins necessary to grow cancer cells [14–16]. One of the undesirable effects of the DOX is damaging normal cells surrounding the malignant tumor and preventing them from uncontrolled proliferation [17]. Another common cancer chemotherapy drug is PAX, a microtubule inhibitor and a member of Taxol anticancer drugs [18, 19]. Co-treatment of PAX and DOX is a common and useful regime in cancer therapy researchs. So that, drug development and side effect reduction of these drugs are desirable in pharmaceutical sciences. The simultaneous release of PAX and DOX has been the subject of many drug studies [20].

One of the strategies to reduce these adverse effects of PAX and DOX is applying smart DDS. A smart delivery system can minimize the damage of this drug to non-cancerous cells of the body and maximize the efficiency of drug use for cancer treatment. Different smart drug delivery systems (DDS) have been designed with various approaches (e.g., pH-responsive, enzyme responsive, redox-responsive, etc.) [21, 22]. Nanotechnology helps experimenters to evolve novel nano-pharmaceutical agents in therapeutic applications [23, 24].

Nevertheless, the functionalized carbon-based nanostructures have newly attracted much attention in biochemical science. SWCNT, as a popular carbon nanostructure, has more applications in nanomedicine [25]. The surface properties of SWCNTs can be modified using dopants and functional groups [26]. The surface of nanomaterials can be functionalized to increase the solubility, biocompatibility, and transport of different biomaterials. The SWCNTs are used as nano-carriers for transporting biomolecules (e.g., proteins, DNA molecules, and medicines). Therapeutic compounds are loaded onto or inside the structures [23].

Targeting and transporting more than one compound simultaneously are other features of interest in drug delivery. Numerous researchers have focused on the potential of carbon nanotubes as carriers for anticancer agents. Carbon-nanotubes have unique physical and chemical properties for drug delivery, enabling them to escape from an immune response. Some of these features are; size, hydrophobicity, geometric shape, surface chemistry and charge [27].

Previous studies have indicated that the carbon nanotubes enter the cell vertically by the endocytosis process [28, 29]. The drug is encapsulated into the nanotube and protected while circulating in the body. Upon getting the target area, the nanotube releases drug, and the encapsulating material erodes and disappears [30, 31]. In spite of their specific characteristics, concerns have transpired apropos’ toxicity of CNTs, as many works have reported pristine nanotube could instigate biological destruction. Karnati et al. conducted a simulation study using the AMBER16 package software. They studied the simultaneous loading and releasing of two drugs. They showed that by functionalizing the carbon nanotube surface, a positive effect could be made on loading and release of drugs [32].

In the current study, functionalized SWCNT nanohybrid with DMAA-TMC co-polymer decreases SWCNT side effects and suggests a safer nano-carrier. Also, this polymer improves the sensitivity of the carrier. From another point of view, an extensive range of investigations have indicated that nanotube development by functionalizing with a carboxylic group improves the applicability in drug delivery pharmacodynamics and pharmacokinetics. In this study, pH-responsive fSWCNT was modified by non-covalent interaction with DMAA-TMC. Thus, we designed a biocompatible functional SWCNT capable of targeted, smart, and selective delivery to cancerous tissues. TMC, as a redesigned polysaccharide-based polymer from chitosan, has an undeniable role in adsorption enhancing in novel nanomedicines [33]. The effect of DMAA-TMC co-polymer on functional SWCNT was appraised as a potential therapeutic system to improve the simultaneous delivery of PAX and DOX in physiological environments with different pH values [34].

The function of pH, carrier size, molecular bonds, and functional groups have been investigated in this work. Previous studies have not investigated the mechanism of DOX-PAX adsorption, penetration, and release from this modified type of CNT using molecular dynamics. The current study aims to introduce modified carbon nanotubes as suitable carriers for the DOX and PAX delivery. Based on the unique aspects of this carrier, this could be a good introduction to the greater investigation of modified carbon nanotubes to load and release antineoplastics.

## Results and discussions

Different loading and release mechanisms of DOX and PAX were studied in two physiological (pH = 7.4) and cancerous (pH = 5.5) conditions to predict cellular penetration, adsorption, and release.

### Drug adsorption in the neutral condition (pH = 7.4)

**Fig. 1:**
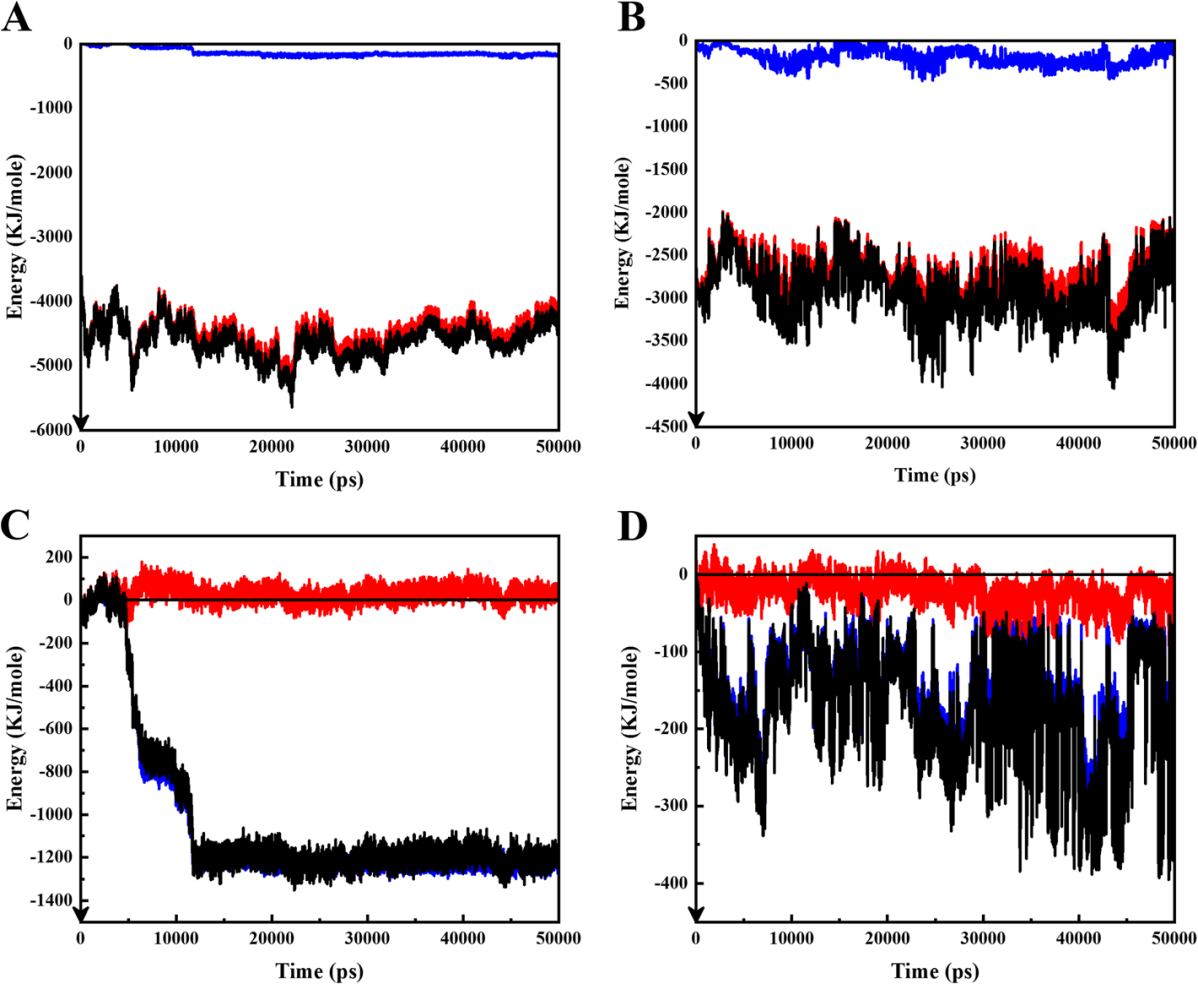
The blue illustration shows the VdW energy, and the red shows electrostatic energy, and the black is for total energy. **A:** represents DOX_f SWCNT interaction, in which VdW force is zero. **B:** represents DOX_DMAA-TMC interaction. **C:** represents interaction of PAX and fSWCNT, in which electrostatic force is zero. **D** represents interaction of PAX and TMC-DMAA. All the figures are under neutral conditions

The interaction of Doxorubicin molecules with the functional SWCNT is shown at Fig. 1A. The blue illustration reveals the VdW energy, and the red points to electrostatic energy, and the black indicates total energy. The electrostatic energy has a significant portion of interaction energy between fSWCNT and DOX at neutral pH. This is thanks to carboxylic acid functional groups charge on carbon nanotube surface. The carboxyl acid group possesses a negative charge (COO^−^) at neutral pH. While in the interactions between PAX and fSWCNT state, electrostatic energy is on the point of zero, and the VdW has a major portion of total interaction energy (Fig. 1C). This is correlative to the hydrophobic intrinsic features of PAX.

Besides, DOX has an amine group, having different charges in the acidic and the neutral environments. In accordance with results, DOX functional groups and fSWCNT with their anonymous charges have strong electrostatic interactions in neutral condition. By considering higher electrostatic energy at normal conditions, more adsorption of drug is expected. In fact, the drug could have a better attachment to the nanotube’s surface at physiological pH values. The carbon nanotube has a vigorous attraction to the DOX, so it is a very suitable carrier for this drug. The following figure (Fig. 1B) displays the attraction between DOX and the copolymer. The VdW force is near to zero, but there is a considerable negative electrostatic energy between drug and  copolymer. Such an interaction is seen in this pH which is owing to the chitosan negative charge. The total negative energy directs a powerful attraction between the medicines and the copolymer.

Figure 1C shows the interaction of paclitaxel with fSWCNT. VdW energy is more pronounced whereas electrostatic energy is on the verge of zero. The SWCNT is functionalized by -COOH functional groups. This has negative charge at physiological pH (COO-) and also no any charge at the acidic (COOH). PAX has no charges in the neutral condition. Correspondingly, electrostatic interaction of PAX and carbon nanotube is near to zero in the physiological condition. VdW contributes considerably to the uptake of PAX on fSWCNT. Figure 1D illustrates the PAX_copolymer interaction. VdW energy displays a more significant amount, and the electrostatic energy is nearing to zero.

Moreover, PAX owns zero charges. Consequently, electrostatic interaction betwixt copolymer and PAX is near to zero in the physiological state. VdW energy has a significant contribution to the attachment of PAX on fSWCNT.

### Investigating hydrogen bond formation

**Fig. 2:**
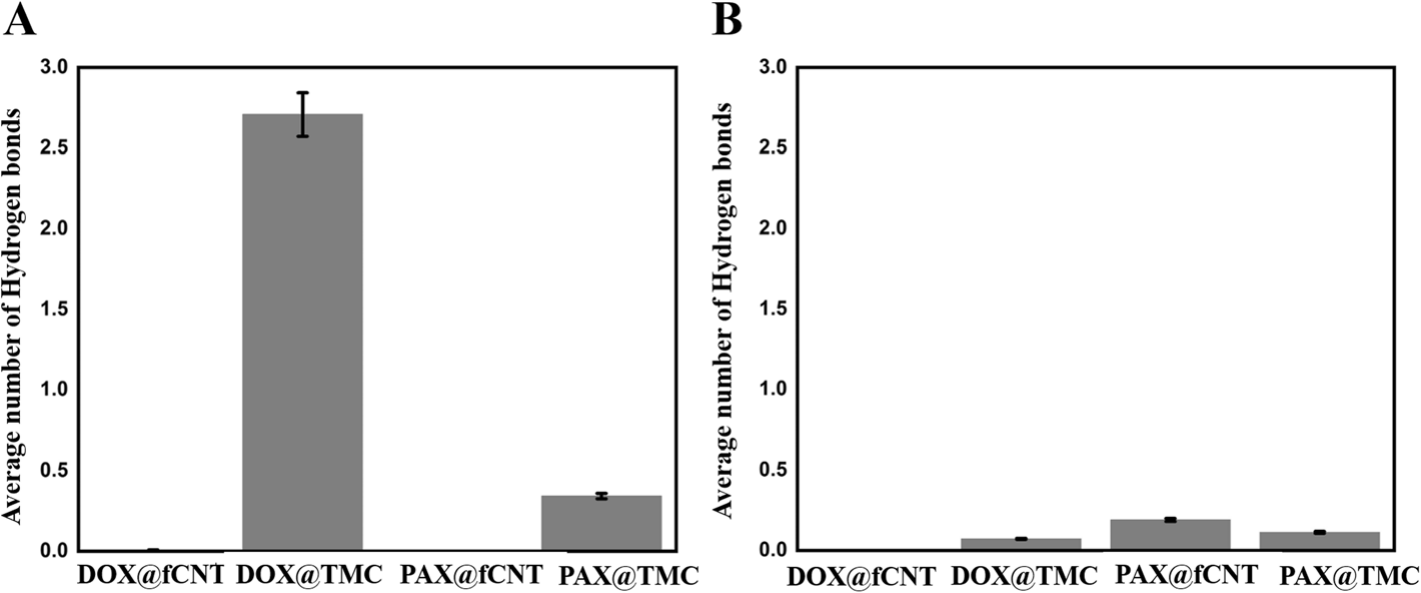
**A:** represents average number of H-bonds. For example, DOX@fSWCNT indicates H-bonds between fSWCNT and DOX at neutral pH, and **B:** represents average number of H-bonds; for example, DOX@fSWCNT indicates H-bonds between fSWCNT and DOX at acidic pH

The hydrogen bond (H-bond) is a significant factor in drug delivery (e.g., loading and release) investigation. The histograms of the H-bonds average number over simulation time between the polymer-polymer and copolymer-drug and CNT-drug for all three simulations are presented in Fig. 2 H-bonds indicate the amount of hydrophilicity property of carriers. Comparing PAX and DOX demonstrate that average number of DOX@DMAA-TMC H-bonds is more than the PAX@DMM-TMC H-bonds, making it stronger. The strength of H-bonds betwixt the copolymer and DOX is relatively high. So, adding the copolymer will be effective in adsorption. However, there are no significant H-bonds between fSWCNT and drugs at neutral pH (Fig. 2A).

**Fig. 3:**
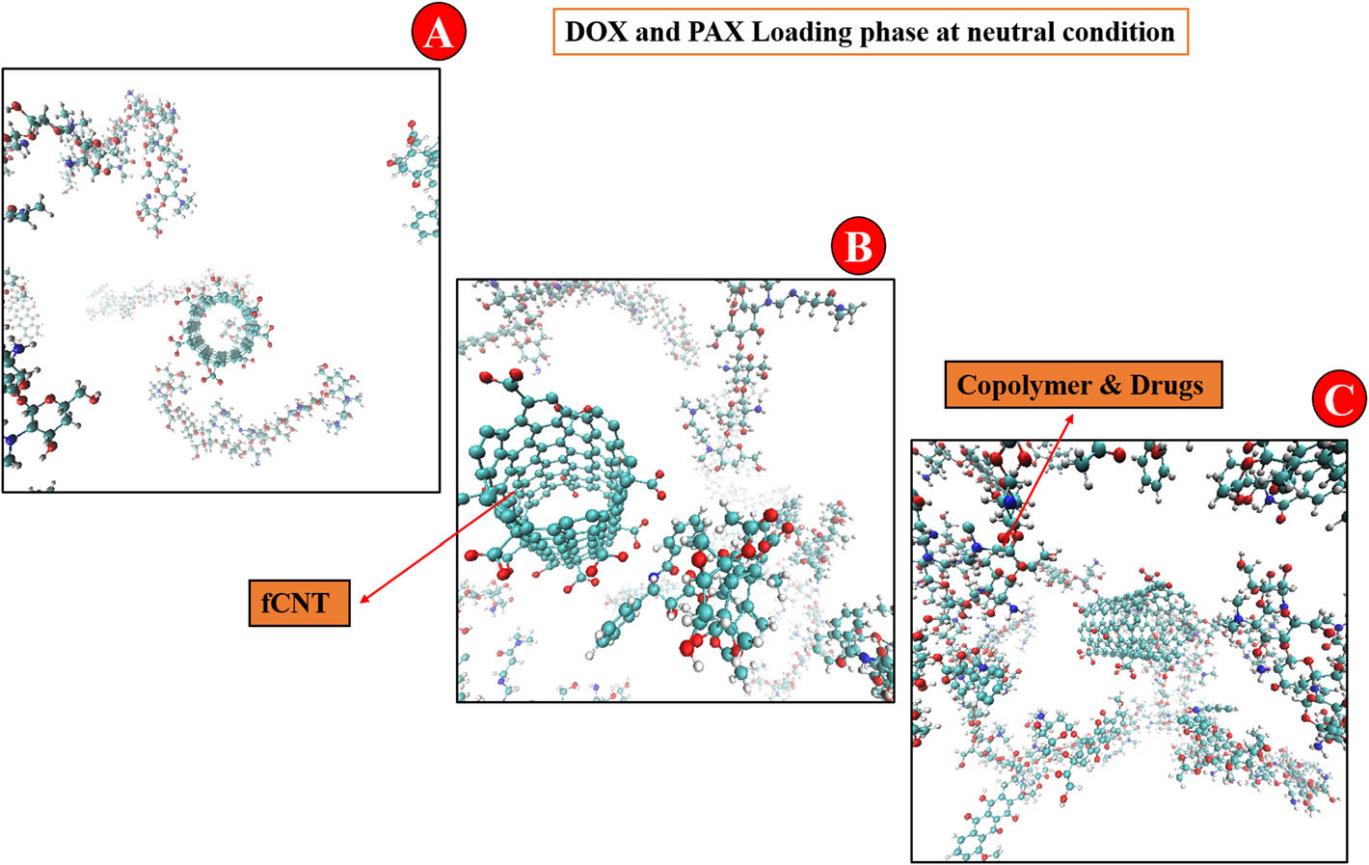
3D schematic DDS for neutral condition at **A:** 0 ns, **B:** 25 ns, and **C:** 50 ns. The figures show the loading simulation during the 50 ns time

Figure 3 illustrates the 3D schematic of DDS at neutral pH. It shows that the adsorption of drugs on carriers increases during the simulation time.

### Drug release in acidic condition (pH = 5.5)

**Fig. 4:**
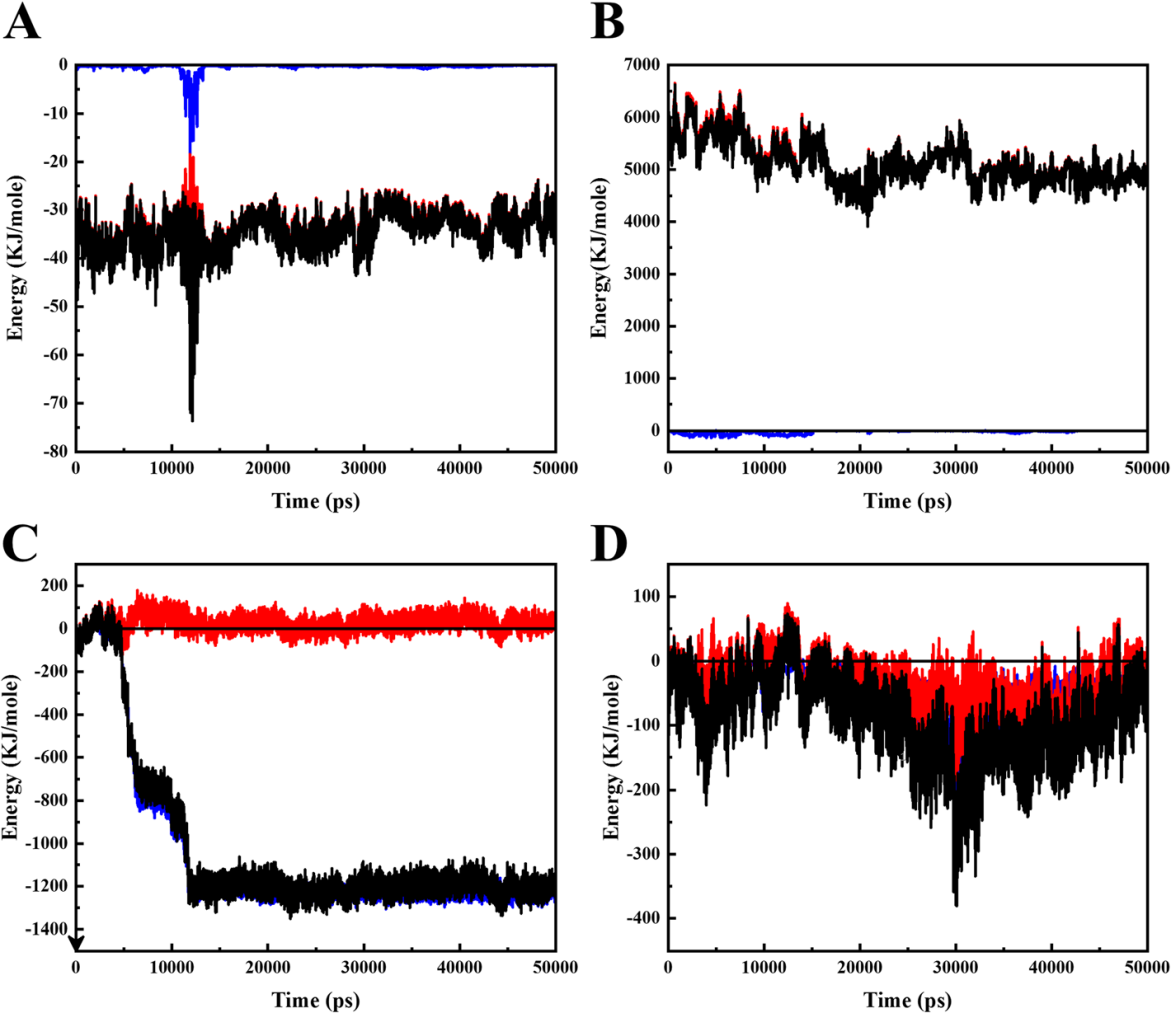
The blue illustration shows the VdW energy, and red shows electrostatic energy, and black is for total energy. **A:** represents the interaction between fSWCNT and DOX which electrostatic is zero. **B:** represents DOX_DMAA-TMC interaction which electrostatic is zero. **C:** represents the interaction between fSWCNT and PAX which VdW is zero. **D:** represents the interaction between TMC-DMAA and PAX. All figures are in acidic condition

The interactions of DOX molecules with fSWCNT nano-carriers are illustrated in Fig. 4. At cancerous pH, electrostatic energy is about zero, and VdW energy offers total interaction energy. The carboxyl acid functional groups functionalize the CNT. The -COOH has negative charge at physiological condition (COO-) and no charge at cancerous pH (as COOH). On the other side of the coin, DOX owns an amine group in the side chain. It comprises a positive charge at cancerous pH (as -NH_3_^+^) and no charge at neutral pH (−NH_2_). As a result, drug functional groups and nanotubes have anonymous and find strong electrostatic interactions in neutral state. However, at acidic pH, fSWCNT charges become zero. Furthermore, electrostatic energy betwixt the fSWCNT and DOX is zero too.

Figure 4B shows the interactions between DOX and degraded polymer molecules. The fascinating fact in the diagram is that in a cancerous condition, the interaction between the antineoplastic and the degraded polymer shows upper zero electrostatic energy. This matter means a loathing between TMC and the drug, which is very useful in the releasing mechanism. Repulsion between TMC and drug causes the better release of drug from TMC and fSWCNT surface.

Doxorubicin has a positive charge in acidic conditions (because of the amine group), and also, after degradation of the copolymer, it has a positive amine group. So, two positively charged molecules repel each other. The presence of chitosan in DDS makes the drug release smart in the cancerous tissues. This is because of the imine group between DMAA and TMC. This bond is sensitive to lower pH values in acidic conditions; this fact facilitates copolymer degradation at this cancerous tissue. By degrading the copolymer, the surface charges of TMC and DMAA are released and play a critical role in releasing drugs in that system.

Figure 4C shows VdW and electrostatic interactions between PAX and fSWCNT. Electrostatic energy is approximately zero, and total is approximately equal to the VdW energy. The loss of electrostatic energy is owing to the lack of charge of carboxyl acid group at cancerous pH. Moreover, PAX has no surface charge. Thus, electrostatic energy between fSWCNT and PAX is zero, and the VdW interaction is weak. The fragile interaction energies cause a better release from the functional SWCNT; this is considered a noteworthy feature for the selected carrier, which is valuable in drug release.

Also, Fig. 4D demonstrates interaction of PAX and degraded polymer in an acidic state. As shown, electrostatic energy and VdW between the chitosan and drug is zero, which helps the drug release. The zero-surface charge of PAX leads to zero electrostatic energy.

Figure 2B demonstrates that DOX is not bound to fSWCNT but has numerous H-bonds with polymer. This figure illustrates the vital role of the polymer in this DDS because TMC forms more hydrophilic carriers through hydrogen bonding formation. CNTs and PAX own high hydrophobicity, which is a fundamental problem for therapeutic carriers. TMC has solved bloodstream blockage by hydrophilizing the complex. The graphs also demonstrate that PAX did not establish eligible hydrogen bonds with fSWCNT, while the same drug with TMC has many.

**Fig. 5:**
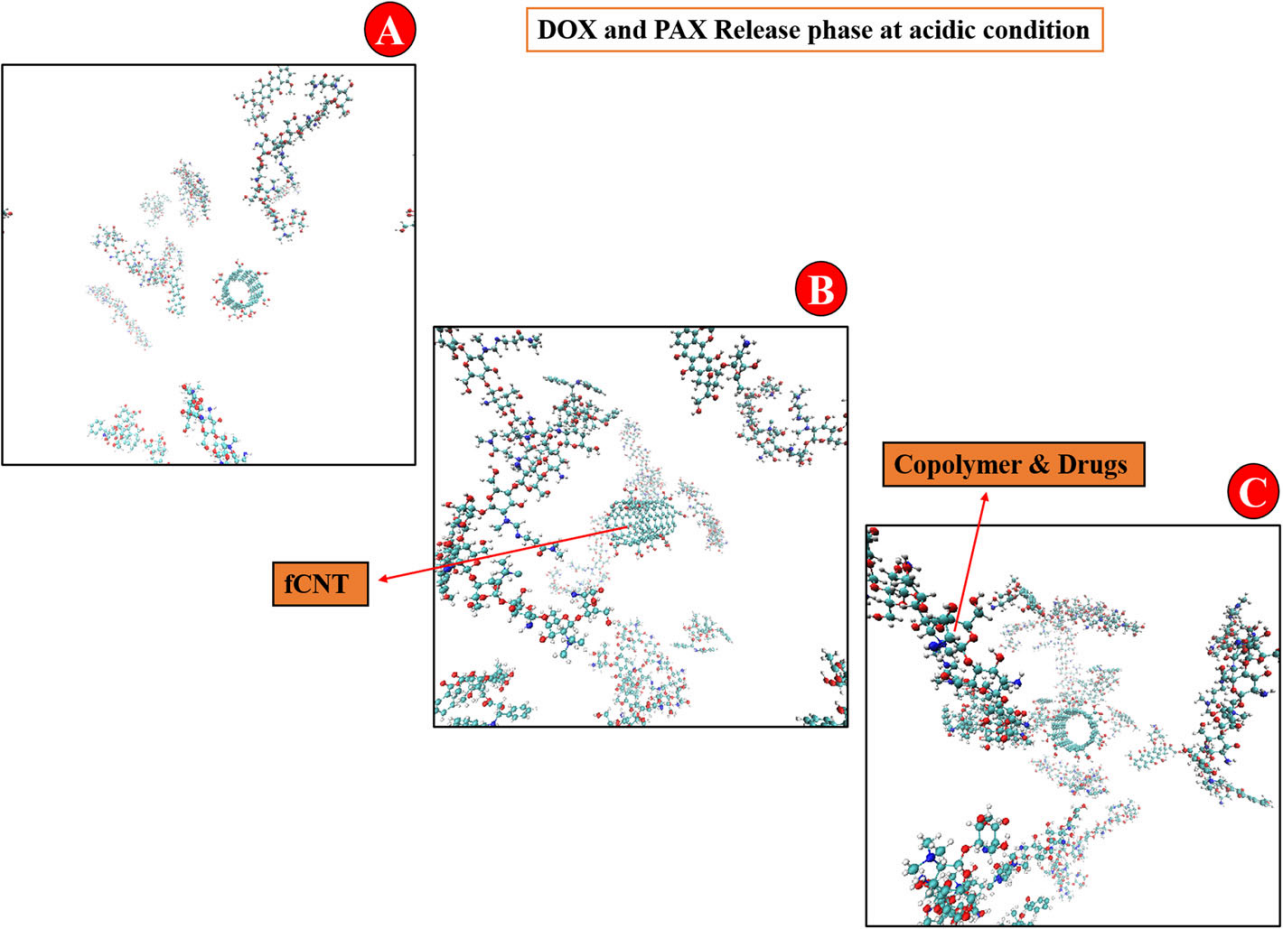
3D schematic DDS for the acidic condition at **A:** 0 ns, **B:** 25 ns, and **C:** 50 ns. The figures show the release simulation during the 50 ns time

Figure 5 indicated the schematic figures of DDS at acidic conditions. This indicated that the distance between molecules improves during the simulation time, and drug release is facilitated.

### Particle size evolution

The radius of gyration (Rg) is one factor empowering the aggregation of molecules and their resizing. The average amount of radius of gyration is presented in Table 1. The values of Rg show the assembly of particles in one region of simulation box. The higher the Rg, the greater the dispersion between the molecules. A valuable gathering of drugs is being formed around the fSWCNT. It means that polymer molecules are grouped in this simulation. DOX also has a higher radius than the PAX, revealing a better aggregation of PAX than DOX. Complexation because of the collection of PAX drugs is more concentrated and stable. Overall, the DDS radius of gyration is shown in Table 1. These data indicate that at acidic conditions, Rg increases. So, the release is better.

**Table 1:** Average of SASA and Rg during the 50,000 ps

	pH = 7.4	pH = 5.5
**Radius of gyration (nm)**	1.81	1.96
**SASA (nm**^**2**^**)**	228.68	235.35

On the contrary, Rg is lower at neutral conditions, which indicates the loading is higher. There is a difference of about 0.15 nm in both neoplastic and physiological conditions at their radius. This difference suggests that the size of molecules is not the same in both cases. In acidic state, because the tendency of molecules and drugs to nanotube is lower than that of molecules under neutral conditions, it has a larger radius of gyration.

By applying ‘gmx sasa’ command, an essential factor is computed. This value indicates the amount of solvent available for the fSWCNT. The higher the amount, the more solvent available to the carrier. Therefore, the distances between the polymer, drugs, and carrier are greater. Furthermore, vice versa is also true. According to Table 1, this value is higher for acidic conditions while lower for neutral conditions. Therefore, in acidic conditions, the distance between the molecules is greater, and drug release increases. However, in neutral conditions, the distance between the molecules is lower, and drug absorption is greater. In another study [35], the amount of SASA has been reported to be higher at higher pH values. So, the chitosan-based structure is more aggregated and condensed.

### Drug diffusion coefficient

**Fig. 6:**
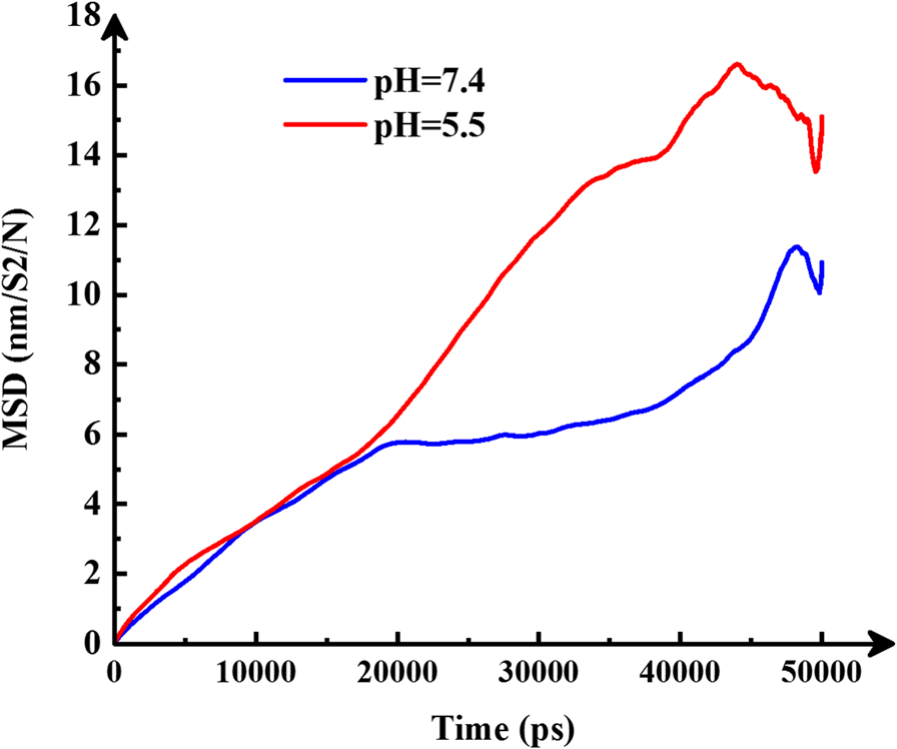
The mean square displacement versus time (ps) for DOX at two conditions. The red line is for acidic conditions, and the blue line is for neutral conditions

The diffusion coefficient (D) is represented by mean square displacement (MSD) curve slope. Figure 6 manifests D of DOX in simulation box. The steric hindrance is less in the acidic microenvironment. Because the adsorption of drug on the functional SWCNT surface is less, so their accumulation is lower than that of neutral state. So, the number of collisions and steric hindrance is less. Eventually, the reduction of the steric hindrance will increase the diffusion coefficient. So, in acidic state, drug diffusion rate is higher (Fig. 6). In a similar study [36], graphene oxide and Doxorubicin-based DDS in three conditions (basic, neutral, and acidic) have been investigated. The average diffusion coefficient (D) for cancerous and physiological have been reported to be 1.97 and 1.27 (10^5 cm^2^/s), respectively. Moreover, MSD for acidic is 0.99 higher than neutral.

### Stability of drug delivery system

**Fig. 7:**
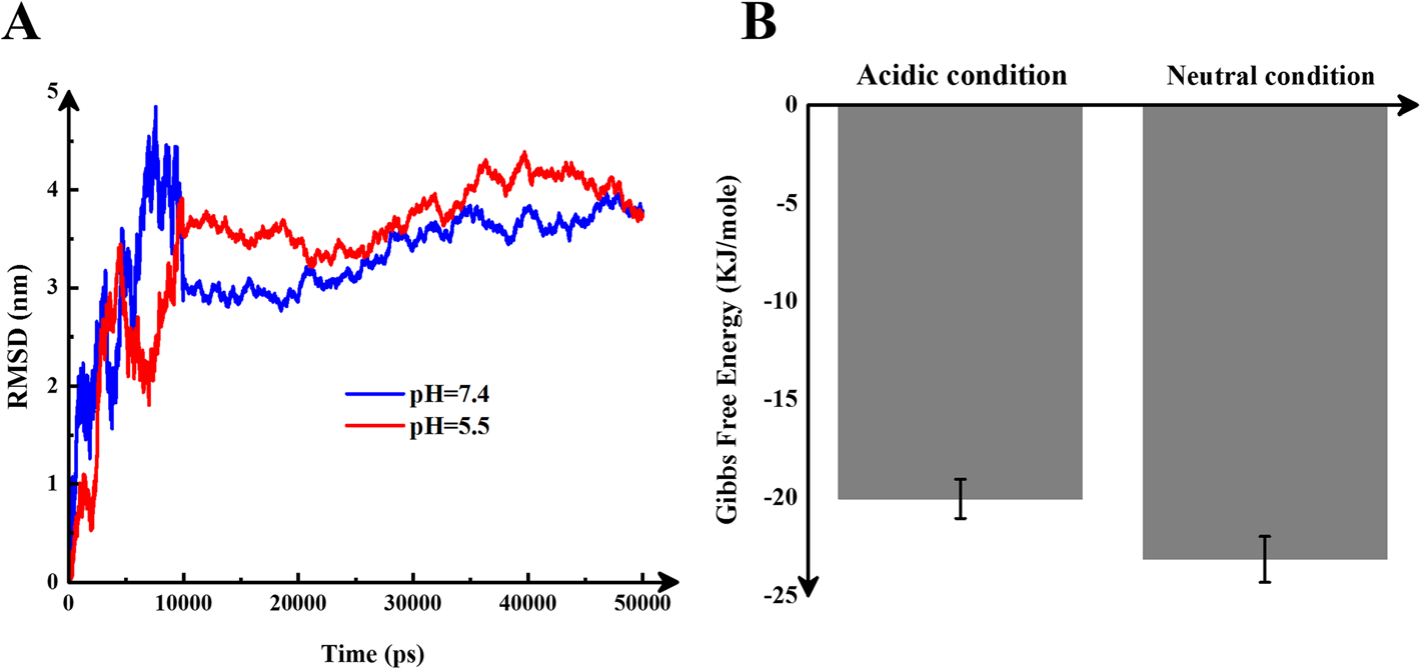
**A:** RMSD values versus time (ps) fluctuation during 50,000 ps. The red line is acidic, and the blue line is for neutral conditions. **B:** Gibbs free energy at acidic and the neutral conditions. DDS at neutral condition is more stable

Root mean square deviation is a suitable criterion for evaluating the simulation system’s stability. The lower the oscillations of this diagram during the simulation time, the greater the stability and equilibrium. Also, its lower value indicates the stability between the two systems being compared. According to Fig. 7A, both graphs have large fluctuations from zero to 10,000 ps. Nevertheless, after 10,000 ps, the fluctuations of both charts have been minimized, and the slope of their variations has been zero. Therefore, from this point to end of simulation, the system is in equilibrium.

In reverse, the blue diagram, related to neutral condition, has a lower average. Therefore, molecules of DDS are more stable in neutral state. RMSD diagrams in the same systems have similar graphs. Nevertheless, there are two critical notes: first is the fluctuation of the diagram be reduced, so the RMSD has a constant graph with low fluctuations. This indicates the equilibrium of simulation systems, and the second is the amount of RMSD. The lower one during the simulation has a more stable system. In a computational study [36], similar to the present work, the RMSD pattern of several systems based on doxorubicin and CNT with different polymer concentrations was drawn. In this study, polymer-free DDS have the lowest RMSD and the most stable state. In contrast, the two systems with 30 and 23 polymers have similar RMSD patterns. Therefore, in the present study, as the molecules and drug delivery system are almost the same, the RMSD pattern is also similar.

To investigate the spontaneity of the simulation system, we calculated Gibbs free energy in both the acidic and neutral states (using umbrella sampling method). A more negative value indicates the stability of the system. According to Fig. 7B, this factor is more negative for normal condition. Therefore, the DDS is more stable in a normal state and is appropriate for loading drugs. The differences between the cancerous and neutral conditions (~ 3 kJ/mol) are related to the position of molecules at both states. In acidic conditions, the tendency of molecules to repel each other is high, but the tendency to compile the molecules with each other is higher at neutral. In other words, the lower Gibbs free energy in the acidic condition indicates that the drug’s affinity to the nanocarrier is ~ 3 kJ/mol lower than in neutral condition, and the drugs are more easily released. So, the energy is more negative in neutral condition.

## Conclusions

Molecular insight of DDS based on carbon nanotubes has been the subject of this study, which has shown that carbon nanotubes can be a suitable nano-carrier for these drugs through the mechanism of pH change. In this computational work, the interactions between DOX and PAX with single-walled nanotubes at different acidic and neutral pHs were investigated. By applying molecular dynamic simulation, the results manifested that the appropriate uptake of the drug occurs at neutral pH (as a normal pH of human blood). From anothr point of fact, its optimal release occurs at an acidic pH (normal pH within cancerous tissue microenvironment). The influence of dimethyl acrylamide trimethyl chitosan in this work was investigated, and it was vindicated that chitosan has a significant function in the DOX release mechanism and PAX uptake. Chitosan also helps to make this carrier more biocompatible. Fantastically, the results revealed that nanotubes and chitosan combinations are useful for simultaneous adsorption and release of cancer chemotherapy drugs. It is suggested that this system can be tested in experimental research by using tissues of living organisms for future prospective works.

## Method

The basis of the molecular dynamics work is the numerical integration of Newton’s equations of motion for every single particle in the system. By applying Newton’s equations of motion, a set of atomic positions is obtained successively. By using molecular dynamics, the state and properties of the system at any later time can be predicted from its current state.

### Molecular dynamics

GROMACS 5.1.2 software was employed for molecular dynamics simulation with OPLS-aa force field (user-friendly and open source). TIP3P was employed as water model. Energy reduction was performed on all 50,000-step simulation systems by steepest descent method to eliminate VdW interactions and form H-bonds between water molecules and other molecules. Further, we gradually raised the system temperature from 0 K to 310 K and for 100 ps time in constant volume; employing the Nose-Hoover algorithm. We balanced the system pressure using Parrinello-Rahman algorithm. We performed Molecular dynamics simulation at 37 °C for 50 ns. We set cut-off distance at 1.2. Particle Mesh Ewald (PME) was used to calculate the electrostatic force.

In order to calculate the permeability coefficient of the drug in the square vector, we calculate the mean displacement of the atoms with ‘r’, and ‘t’ represents time. The following formula shows the mean square of the displacement:

1 $$ \mathrm{MSD}=\left\langle {\left[\mathrm{r}\left(\mathrm{t}\right)-\mathrm{r}(0)\right]}^2\right\rangle =\frac{1}{\mathrm{t}}\sum \limits_{\mathrm{t}={\mathrm{t}}_0}^{\mathrm{t}}{\left[\mathrm{r}\left(\mathrm{t}\right)-\mathrm{r}(0)\right]}^2 $$

After calculating the MSD, the diffusion coefficient (D) for a three-dimensional system can be derived from Einstein’s relation.

2 $$ \mathrm{D}=\frac{1}{6}\underset{\mathrm{t}\to \infty }{\lim}\frac{\mathrm{MSD}}{\mathrm{t}} $$

### Carbon nanotubes simulation

TubeGen Online web server (https://turin.nss.udel.edu/research/tubegenonline.html) was used to make single-walled carbon nanotube molecules. Carboxylated functional groups were added to the CNT surface in both deprotonated and protonated states. Optimized Potentials for Liquid Simulation-all atom (OPLS-aa) force field was defined for parametrization. Lenard-Jones models and Columbian potentials algorithms were used to reckon non-covalent interactions such as VdW and electrostatic.

## Data Availability

The datasets used and/or analyzed during the current study are available from the corresponding author on reasonable request.

## Abbreviations

OPLS-aa: Optimized Potentials for Liquid Simulation-all atom
PME: Particle Mesh Ewald
MSD: Mean square of the displacement
DMAA-TMC: Dimethyl Acryl amide-trimethyl chitosan
DOX: Doxorubicin
PAX: Paclitaxel
fSWCNT: Functionalized single-walled carbon nanotube
RDF: Radial distribution function
DDS: Drug delivery system
VdW: Van der Waals
